# LRRC25 plays a key role in all-trans retinoic acid-induced granulocytic differentiation as a novel potential leukocyte differentiation antigen

**DOI:** 10.1007/s13238-017-0421-7

**Published:** 2017-05-23

**Authors:** Weili Liu, Ting Li, Pingzhang Wang, Wanchang Liu, Fujun Liu, Xiaoning Mo, Zhengyang Liu, Quansheng Song, Ping Lv, Guorui Ruan, Wenling Han

**Affiliations:** 1 Department of Immunology, School of Basic Medical Sciences, Key Laboratory of Medical Immunology (Ministry of Health), Peking University Health Science Center, Beijing, 100191 China; 2 Peking University Center for Human Disease Genomics, Beijing, 100191 China; 3 Institute of Hematology, Peking University People’s Hospital, Beijing, 100044 China

**Keywords:** LRRC25, differentiation antigen, granulocytic differentiation, ATRA, AML

## Abstract

Leukocyte differentiation antigens (LDAs) play important roles in the immune system, by serving as surface markers and participating in multiple biological activities, such as recognizing pathogens, mediating membrane signals, interacting with other cells or systems, and regulating cell differentiation and activation. Data mining is a powerful tool used to identify novel LDAs from whole genome. LRRC25 (leucine rich repeat-containing 25) was predicted to have a role in the function of myeloid cells by a large-scale “omics” data analysis. Further experimental validation showed that LRRC25 is highly expressed in primary myeloid cells, such as granulocytes and monocytes, and lowly/intermediately expressed in B cells, but not in T cells and almost all NK cells. It was down-regulated in multiple acute myeloid leukemia (AML) cell lines and bone marrow cells of AML patients and up-regulated after all-trans retinoic acid (ATRA)-mediated granulocytic differentiation in AML cell lines and acute promyelocytic leukemia (APL; AML-M3, FAB classification) cells. Localization analysis showed that LRRC25 is a type I transmembrane molecule. Although ectopic LRRC25 did not promote spontaneous differentiation of NB4 cells, knockdown of LRRC25 by siRNA or shRNA and knockout of LRRC25 by the CRISPR-Cas9 system attenuated ATRA-induced terminal granulocytic differentiation, and restoration of LRRC25 in knockout cells could rescue ATRA-induced granulocytic differentiation. Therefore, LRRC25, a potential leukocyte differentiation antigen, is a key regulator of ATRA-induced granulocytic differentiation.

**Electronic supplementary material:**

The online version of this article (doi:10.1007/s13238-017-0421-7) contains supplementary material, which is available to authorized users.

## INTRODUCTION

LDAs are a series of glycoproteins and glycolipids attached to or inserted into the membranes of leukocytes, major cells of the immune system that are involved in hematopoietic malignancies. LDAs play important roles in the immune system. They serve as surface markers of immune cells and participate in multiple biological activities, such as recognizing pathogens, mediating membrane signals and interaction with other cells or systems, and regulating cell differentiation and activation (Li et al., 2015). Moreover, many LDA-related drugs have been developed, such as anti-CTLA-4 and anti-PD-1 (Ma et al., 2016). Therefore, identification of novel LDAs that are differentially expressed on various immune cells is needed. Multi-omics analyses have generated abundant data, such as gene expression data from the Immunological Genome Project (ImmGen) (Heng and Painter, 2008) and the transcriptomic data collection from human and mouse immune cells (Wang et al., 2015a, b). This has made it feasible and convenient to systematically identify novel differentiation antigens. Using bioinformatics analysis, we identified LRRC25 (leucine rich repeat-containing 25) as a leukocyte differentiation antigen with a characteristic expression profile and further assessed the function of this molecule.

*LRRC25* is located at human chromosome 19p13.11, which is a leukocyte receptor enriching cluster. The deduced polypeptide of human LRRC25 is composed of 305 amino acids. The predicted protein has 4 leucine-rich repeats at the N-terminus, which may be associated with host-pathogen interactions, and several potential N-linked glycosylation sites (Kedzierski et al., 2004). At the C-terminus, there are two tyrosine-based motifs, one for interaction with phosphatidylinositol-3 (PI3) kinase (YENM) and one that is a “closet” ITIM (immunoreceptor tyrosine-based inhibitory motif, S/I/V/LxYxxI/V/L) (Barrow and Trowsdale, 2006)-within-an-ITAM (immunoreceptor tyrosine-based activation motif, YxxI/L(7/8)YxxI/L) (Rissoan et al., 2002). The “closet” ITIM-within-an-ITAM could mediate inhibitory signaling under conditions of partial ITAM phosphorylation, and several ITAM- and ITIM-encoding proteins are crucial for the development of hematopoietic cells (Barrow and Trowsdale, 2006). LRRC25, also known as MAPA (monocyte and plasmacytoid-activated protein), was reported to be expressed in dendritic cells (DCs), granulocytes, monocytes, and B cells instead of T cells, the expression level of LRRC25 in B cells was obviously lower than that in granulocytes or monocytes, and it was down-regulated in CD40-activated monocyte-derived DCs (MDDCs), activated granulocytes, and B cells (Rissoan et al., 2002). One expressed SNP (rs6512265) of LRRC25 was associated with malaria infection (Idaghdour et al., 2012), and LRRC25 expression was one of the most relevant parameters for describing Vitamin D responsiveness *in vivo* (Vukic et al., 2015). However, the function of LRRC25 is unclear thus far.

Many LDAs have been reported to be involved in the pathogenesis and development of hematopoietic malignancies. Certain antigens are used as markers for diagnosis, classification, and risk stratification and therapeutic targets (Li et al., 2015). The vast majority of APL cases are characterized by a balanced reciprocal translocation between chromosomes 15 and 17, resulting in the fusion of the promyelocytic leukemia (*PML*) gene and retinoic acid receptor α (*RARα*) (Wang and Chen, 2008; De Braekeleer et al., 2014). APL prognosis has improved significantly since the introduction of ATRA, a front-line drug for treatment of APL (Wang and Chen, 2008). The fusion protein PML/RARα forms homodimers and binds to a set of retinoic acid response elements (RAREs), recruits corepressor (CoR) proteins, and represses the transcriptional expression of target genes essential for granulocytic differentiation (Wang and Chen, 2008). In addition to affecting the transcriptome, PML/RARα initiates leukemia by altering the methylome (Gaillard et al., 2015). ATRA is used in APL differentiation therapy; it converts PML/RARα from a transcriptional repressor to a transcriptional activator and induces its proteolysis (Dos et al., 2013; Nitto and Sawaki, 2014). However, the downstream regulators, which may include LDAs, remain unclear.

Here, we report that LRRC25, a type I transmembrane molecule, is a potential leukocyte differentiation antigen and regulates myeloid cell differentiation. In addition, LRRC25 was down-regulated in AML cells and highly expressed in primary myeloid cells. It was up-regulated after ATRA-induced differentiation of AML cells. Knockdown or knockout of LRRC25 inhibited ATRA-induced granulocytic differentiation, and restoration of LRRC25 in knockout cells rescued ATRA-induced granulocytic differentiation. Our results identified LRRC25 as a novel potential leukocyte differentiation antigen, which is a key regulator of ATRA-induced granulocytic differentiation.

## RESULTS

### LRRC25 is a potential leukocyte differentiation antigen identified by bioinformatics analysis

**Figure 1:**
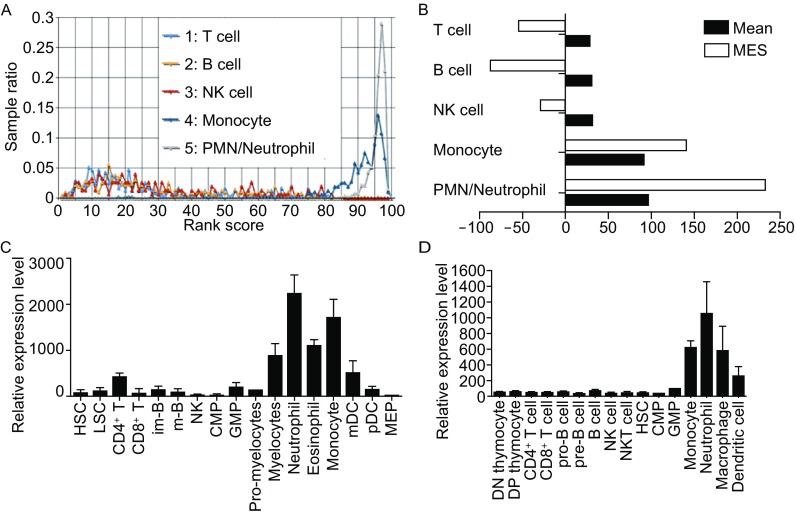
**LRRC25 is a potential leukocyte differentiation antigen identified by bioinformatics analysis**. (A) Rank-based expression (RBE) curve of LRRC25 shows it was lowly expressed in lymphocytes including T cells, B cells, and NK cells, and highly expressed in monocytes and PMNs/neutrophils. Bioinformatics analysis was based on the ImmuSort Database (http://immusort.bjmu.edu.cn). (B) Histogram combined the marker evaluation score (MES) and mean shows that LRRC25 may be a potential biomarker, particularly for PMNs and monocytes. (C) RNA-seq data revealed that LRRC25 was poorly expressed during development of human lymphocytes but highly expressed in terminal stages of myeloid cells. (D) Expression of LRRC25 during development of lymphocytes and myeloid cells in mouse according to the ImmGen database shows similar expression pattern with that in human

We built the ImmuSort database, which provides a convenient way to view global differential gene expression across thousands of experimental conditions in immune cells. Potential biomarkers can also be analyzed using the database by the combination of average rank score (ARS) and marker evaluation score (MES) (Wang et al., 2015). The ARS (in a scale of percentile) reflects gene expression intensity, while the MES reflects the variability of ARS among different immune cells. Genes with a very small or large ARS are likely to have lower gene expression variation under diverse conditions and can be used as negative or positive marker candidates, respectively (Wang et al., 2015; Wang et al., 2016). After searching the ImmuSort database, we found that human LRRC25 was highly expressed in polymorphonuclear leukocytes (PMNs, predominantly derived from neutrophils in the database) and monocytes, moderately expressed in macrophages and DCs, but poorly expressed in lymphocytes, such as T cells, B cells, and NK cells (Fig. 1A). The ARSs of LRRC25 in PMNs, monocytes, monocyte-derived macrophages (MDMs), MDDCs, alveolar macrophages, and tissue resident DCs were 95.98, 90.19, 81.32, 72.9, 67.52, and 64.87, respectively, while they were 31.86, 31.22, 29.67, and 30.38 in CD4^+^ T cells, CD8^+^ T cells, B cells, and NK cells. These results indicated that LRRC25 had significantly increased expression in myeloid cells compared to that in lymphocytes, suggesting that it may be a potential myeloid biomarker, particularly for PMNs and monocytes, as determined by the large MES values of 233.27 in PMNs and 141.9 in monocytes (Fig. 1B).

The ImmuSort database also revealed that the ARS of LRRC25 in blood hematopoietic stem cells (HSCs) and bone marrow HSCs was 38.58 and 35.94 respectively, indicating low expression in these cells. Therefore, we further analyzed the expression of LRRC25 during myeloid and lymphocyte development. As shown in Figure 1C, with the development of myeloid cells, the expression levels of LRRC25 increased, but no apparent change in expression was observed in the process of lymphocyte development. Similar results were also observed in mouse immune cells (Fig. 1D), further supporting the role of LRRC25 as a leukocyte differentiation antigen that regulates myeloid cell development.

In addition to the above data sets from microarray platforms, RNA-seq data also revealed that LRRC25 was poorly expressed in HSCs, lymphocytes, and their precursors but highly expressed in myeloid cells. For example, based on the GSE69239 data set, the FPKMs (fragment per kilobases of exon model per million reads) of LRRC25 in HSCs, LMPPs (lymphoid-primed multipotent progenitors), CLPs (common lymphoid progenitors), and BCPs (B cell-committed progenitors) were 1.26, 2.62, 0.03, and 0.02, respectively. In immature double-positive thymocytes and mature CD4^+^ and CD8^+^ T cells, the FPKMs were 0.14, 0.06, and 0, respectively. Another data set, GSE25133, revealed that the RPKMs (reads per kilobases per million reads) of LRRC25 in CD4^+^ T cells, CD8^+^ T cells, and granulocytes were 1.35026, 0.82696, and 112.718, respectively. Therefore, both array- and sequence-based platforms indicated that LRRC25 was highly expressed in myeloid cells.

### LRRC25 is down-regulated in myeloid leukemia cells, highly expressed in mature myeloid cells, and up-regulated during ATRA-induced granulocytic differentiation

**Figure 2:**
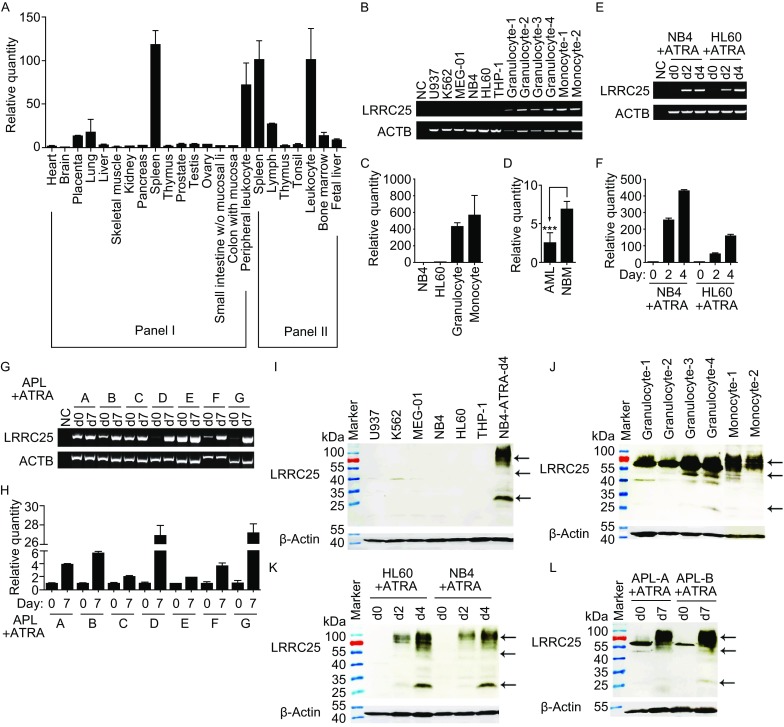
**LRRC25 was down-regulated in myeloid leukemia cells, highly expressed in mature myeloid cells and up-regulated during ATRA-induced granulocytic differentiation**. (A) Real-time PCR analysis of LRRC25 expression in multiple tissues/cells using two MTC^TM^ cDNA panels from Clontech shows that LRRC25 is highly expressed in spleen and leukocytes. The relative LRRC25 mRNA level was shown as a ratio to that in the heart. (B and C) Semi-quantitative PCR and real-time PCR analysis of LRRC25 expression in several myeloid leukemia cell lines and primary myeloid cells including granulocytes and monocytes from healthy donors. Granulocytes fraction and peripheral mononuclear cells (PBMCs) were isolated from peripheral blood by Ficoll-Hypaque density gradient centrifugation. Granulocytes were obtained after lysing red cells and verified by CD11b expression, while PBMCs were cultured for 2–3 h and adherent cells were collected as monocytes and verified by expression of CD14. NC represents negative control. Quantification of LRRC25 was shown as a ratio to mRNA expression in HL60 cells. Data of three donors is shown as mean ± SEM. (D) Real-time PCR analysis shows LRRC25 is down-regulated in bone marrow of AML patients compared with that in normal bone marrow (NBM). *n* (NBM) = 27, *n* (AML) = 32. Error bar represents SEM. **P < 0.01. (E and F) Semi-quantitative PCR and real-time PCR analysis show LRRC25 was up-regulated in ATRA-induced granulocytic differentiation of AML cell lines. Quantification of LRRC25 in each cell line was shown as a ratio to mRNA expression in the un-induced cells (d0). NC represents negative control. Data in triplicates was calculated and error bar represents SD. (G and H) Semi-quantitative PCR and real-time PCR analysis show LRRC25 was up-regulated in ATRA-induced granulocytic differentiation of APL bone marrow cells. Quantification of LRRC25 in each patient was shown as a ratio to mRNA expression in the un-induced samples (d0). NC represents negative control. Data in triplicates was calculated and error bar represents SD. (I–L) Western blot analysis shows expression pattern of LRRC25 on protein level, β-actin was used as a loading control: (I) LRRC25 was poorly expressed in myeloid leukemia cell lines, ATRA treated NB4 samples were used as a positive control. (J) LRRC25 was highly expressed in primary granulocytes and monocytes, which were isolated as indicated previously. (K) LRRC25 was up-regulated in ATRA-induced granulocytic differentiation of AML cell lines. (L) LRRC25 was up-regulated in ATRA-induced granulocytic differentiation of APL bone marrow cells

To investigate the expression profile of LRRC25, we performed real-time PCR to quantify the expression of LRRC25 using MTC^TM^ panels. As shown in Figure 2A, LRRC25 was highly expressed in the immune system, with high expression levels in the spleen and peripheral leukocytes. Then, we detected its expression in several myeloid leukemia cell lines and mature myeloid cells, including granulocytes and monocytes by RT-PCR. LRRC25 was undetectable/poorly expressed in myeloid leukemia cell lines, including U937, K562, MEG-01, NB4, HL60, and THP-1 cells, while it was highly expressed in mature myeloid cells, such as granulocytes and monocytes (Fig. 2B and 2C). To further explore the expression of LRRC25 in primary myeloid leukemia cells, we collected samples of bone marrow cells (27 normal and 32 AML) and detected the expression of LRRC25 by real-time PCR. Figure 2D shows that LRRC25 was down-regulated in AML cells.

ATRA is one of the front-line clinical drugs used to treat APL (AML-M3, FAB classification) (Cicconi and Lo-Coco, 2016). NB4 (M3) and HL60 (M2) cells could differentiate into granulocytes following ATRA treatment (Nishioka et al., 2009). To investigate the expression of LRRC25 in the process of granulocytic differentiation, we treated NB4 and HL60 cells with ATRA and detected the expression of LRRC25 by PCR. As shown in Figure 2E and 2F, LRRC25 was up-regulated during granulocytic differentiation in NB4 and HL60 cells. To further explore the expression of LRRC25 in granulocytic differentiation, we treated APL bone marrow cells of 7 patients with ATRA for a week and detected expression of LRRC25 using PCR. Figure 2G and 2H show that LRRC25 was up-regulated in granulocytic differentiation of the bone marrow cells in all 7 patients. Up-regulated expression of CD11b as determined by flow cytometry indicated successful polarization of NB4 and HL60 cells (Jasek et al., 2008) and APL bone marrow cells (Fig. S1).

To confirm expression of LRRC25 at the protein level and determine its location, we prepared rabbit polyclonal antibody and mouse monoclonal antibodies against LRRC25. As shown in Figure S2A, the prokaryotic proteins GST-LRRC25ic and GST were generated, and GST-LRRC25ic was used as an immunogen to prepare rabbit anti-human LRRC25 polyclonal antibody. Finally, sera in one rabbit could recognize intrinsic LRRC25 expressed in ATRA-treated NB4 and HL60 cells (Fig. S2B). Three specific bands could be observed, i.e., a band between 25 kDa and 35 kDa, which should be the monomer of LRRC25 without a signal peptide (predicted to be 31 kDa); a band between 40 kDa and 55 kDa, which may be glycosylation-modified LRRC25 or complex; and a band covering a wide range near 70 kDa, which may be oligomer or complex with associated molecules. Moreover, the rabbit anti-human LRRC25 antibody could be used for immunoprecipitation in addition to Western blot analysis (Fig. S2C). We also prepared the eukaryotic proteins Fc and LRRC25ec-Fc (Fig. S2D) and generated mouse monoclonal antibodies against LRRC25ec with LRRC25ec-Fc as an immunogen. These monoclonal antibodies were used for flow cytometry and immunofluorescence analyses. Western blots confirmed that LRRC25 is down-regulated in myeloid leukemia cells while highly expressed in granulocytes and monocytes, and up-regulated after induction to granulocytes in HL60, NB4, and APL bone marrow cells at the protein level (Fig. 2I–L).

### LRRC25 is a type I membrane molecule

**Figure 3:**
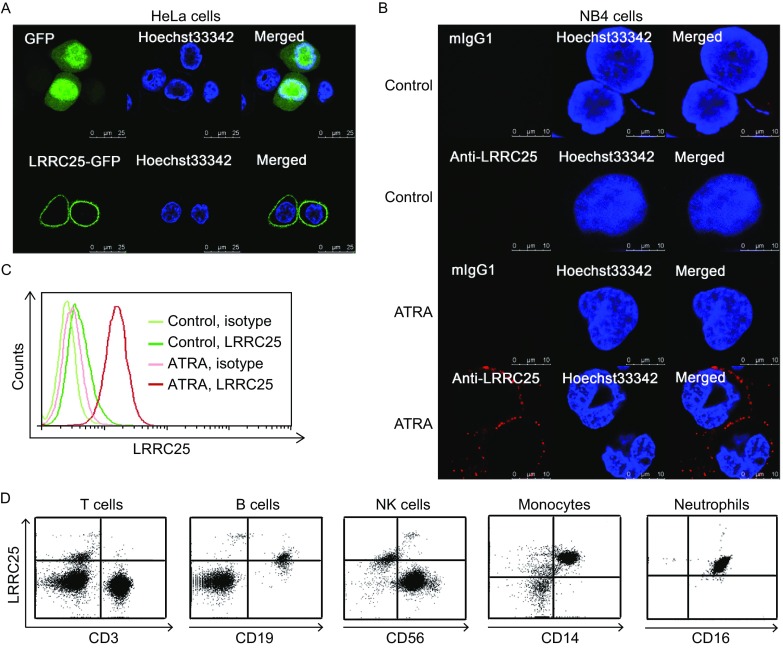
**LRRC25 was located on the plasma membrane and was a type I membrane molecule**. (A) LRRC25-GFP was located on plasma membrane of HeLa cells. (B) A positive fluorescence signal on plasma membrane of ATRA-induced NB4 cells was detected, when cells were not permeabilized and the primary antibody for immunofluorescence was mouse monoclonal antibody against the N-terminus of LRRC25, suggesting N-terminus is outside the plasma membrane. (C) LRRC25 was lowly expressed in control-NB4 cells while it was up-regulated in ATRA-induced NB4 cells. Mouse monoclonal antibody against the N-terminus of LRRC25 was used, and the cells used for flow cytometric analysis were not permeabilized, suggesting N-terminus is outside the plasma membrane, and also the antibody recognized a specific staining. (D) Unpermeabilized cells were used for flow cytometry analysis with mouse anti-human N-terminus of LRR25. Leukocytes were isolated from fresh human peripheral blood by Ficoll-Hypaque density gradient centrifugation, and coexpression of LRRC25 and lineage markers was determined by flow cytometry. Lymphocytes were gated on base of forward and side scatter. CD19^+^ B cells and CD56^+^ NK cells were selected by gating CD3^−^ cells. CD14^+^ monocytes were gated on base of forward and side scatter. Granulocytes were gated on base of forward and side scatter and CD11b expression, neutrophils were selected by gating CD11b^+^ cells. CD3^+^ T cells and most of NK cells did not express LRRC25, B cells expressed low/intermediate level of LRRC25, while monocytes and neutrophils expressed high level of LRRC25. Quadrants were set on base of isotype staining. Data shown is representative of at least three healthy donors

LRRC25 is predicted to be a type I membrane protein (http://www.cbs.dtu.dk/services/TMHMM/). Initially, we investigated localization of LRRC25-GFP in HeLa cells by confocal microscopy and found that LRRC25-GFP localized to the cell membrane, while GFP alone showed diffuse distribution in HeLa cells (Fig. 3A). To confirm that the N-terminus of LRRC25 is extracellularly localized, we used a mouse monoclonal antibody against the N-terminus of LRRC25 to perform immunofluorescence staining of ATRA-induced NB4 cells, which expressed intrinsic LRRC25, by confocal microscopy. As shown in Figure 3B, LRRC25 in ATRA-induced NB4 cells could be recognized without permeabilization, and it was localized at the cell membrane, while no fluorescence signals could be observed in isotype control groups or control NB4 cells, which expressed no LRRC25.

We used the monoclonal antibody against the N-terminus of LRRC25 to perform flow cytometric analysis in ATRA-treated NB4 cells without permeabilization, and found a positive signal only in treated cells (Fig. 3C), suggesting this monoclonal antibody recognized a specific staining. We also used this monoclonal antibody to detect the expression of LRRC25 in peripheral leukocyte subsets including T cells, B cells, NK cells, monocytes, and neutrophils without permeabilization. As shown in Figure 3D, LRRC25 was highly expressed in both CD11b^+^CD16^+^ neutrophils and CD14^+^ monocytes. In contrast, CD3^+^ T cells and most of CD3^−^CD56^+^ NK cells did not express LRRC25. CD3^−^CD19^+^ B cells showed low/intermediate expression of LRRC25 in all three healthy donors, which is consistent with previously reported in the Blood paper (Rissoan et al., 2002).

Given that the epitope recognized by the mouse anti-LRRC25 monoclonal antibody is in the N-terminus, LRRC25 is a type I membrane protein with the N-terminus localized outside the cell membrane.

### Ectopic expression of LRRC25 did not affect proliferation and differentiation of AML cells

**Figure 4:**
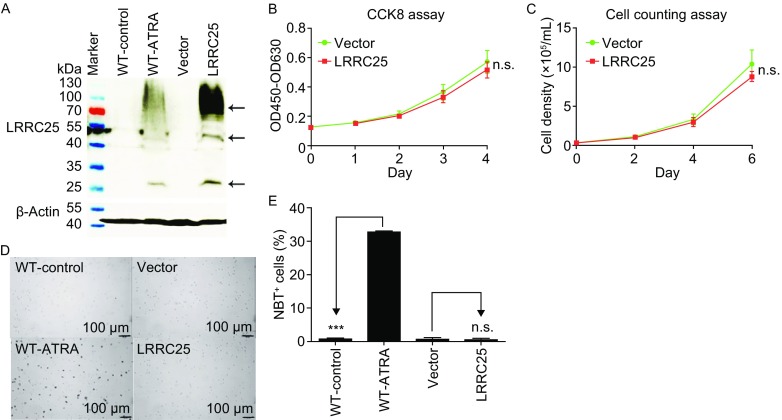
**Ectopic LRRC25 could neither affect proliferation nor promote spontaneous differentiation of NB4 cells**. (A) Verification of ectopic LRRC25 in NB4 cells by Western blot. (B and C) CCK8 (Cell Counting Kit-8) assay and cell counting assay show ectopic LRRC25 did not affect NB4 cells proliferation. Student’s *t* test was used for statistical analysis of data at each time point. Error bar represents SD. n.s. represents no significance. (D) Nitroblue tetrazolium reduction (NBT) assay shows ectopic LRRC25 did not promote spontaneous differentiation of NB4 cells. (E) Quantification of data shown in (D). Student’s *t* test was used for statistical analysis of data. Error bar represents SD. ***P < 0.001. n.s. represents no significance

Cell differentiation is orchestrated with controlled proliferation of progenitor cells (De Kouchkovsky and Abdul-Hay, 2016). Initially, we investigated whether ectopic LRRC25 affected proliferation and promoted spontaneous differentiation of myeloid leukemia cells. LRRC25 was restored in NB4 cells (Fig. 4A). CCK8 assays and cell counting assays showed no significant difference in proliferation between the ectopic LRRC25 group and control group, suggesting that ectopic expression of LRRC25 did not affect proliferation of NB4 cells (Fig. 4B and 4C). The NBT reduction assay is a standard test to evaluate granulocytic differentiation, as differentiated cells can produce superoxide which can be measured by the ability to reduce NBT (Nishioka et al., 2008). As shown in Figure 4D and 4E, over 30% of ATRA-treated NB4 cells reduced NBT, while no ATRA-treated NB4 cells overexpressing LRRC25 could reduce NBT, indicating that ectopic expression of LRRC25 could not promote spontaneous differentiation of NB4 cells.

### Knockdown or knockout of LRRC25 impaired ATRA-mediated granulocytic differentiation which could be rescued by LRRC25 restoration

**Figure 5:**
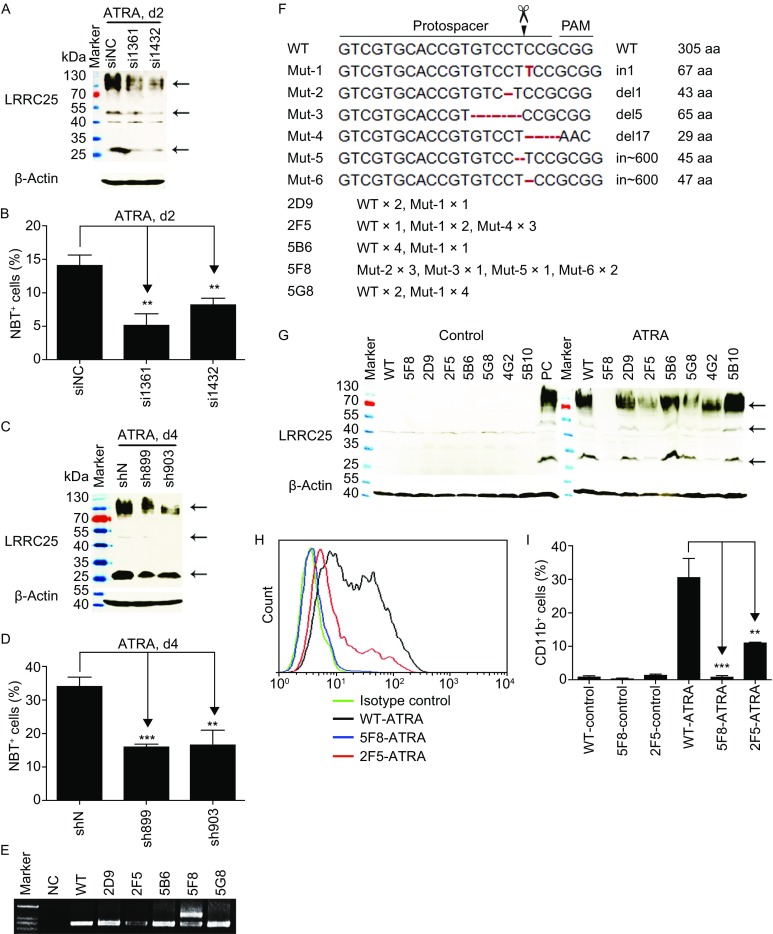

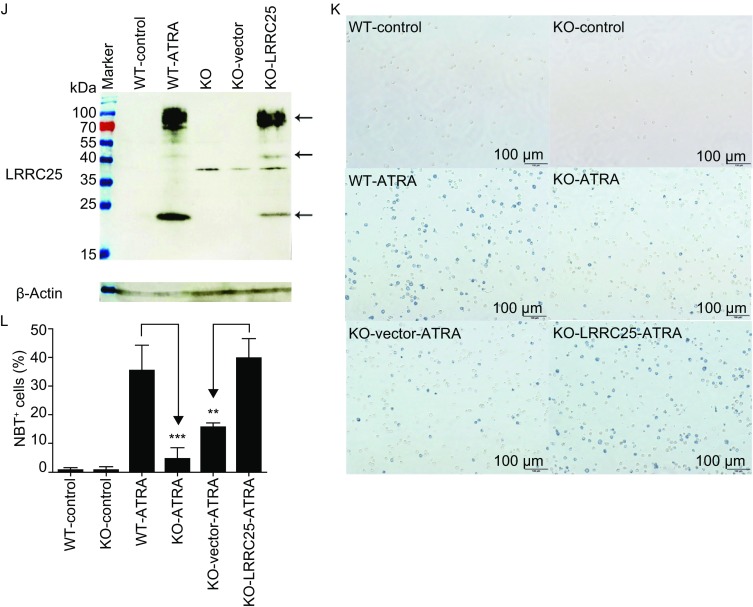
**Knockdown or knockout LRRC25 impaired ATRA-induced granulocytic differentiation which could be rescued by LRRC25 restoration**. (A) Western blot shows LRRC25 was knocked down by si1361 and si1432 compared with that by non-silencing control siRNA (siNC). (B) NBT assay shows decreased ratio of NBT^+^ cells by siLRRC25 knockdown in NB4 cells. (C) Western blot shows LRRC25 was knocked down by sh899 and sh903 compared with that by non-silencing control shRNA (shN). (D) NBT assay shows decreased ratio of NBT^+^ cells by shLRRC25 knockdown in NB4 cells. (E) Agarose gel electrophoresis shows PCR products containing the gRNA targeting site, NC indicated negative control. (F) Sanger sequencing analysis shows different mutations by CRISPR-Cas9 targeting. (G) Western blot shows 5F8 was one cell clone with LRRC25 knockout. (H) Histogram of flow cytometry shows CD11b expression decreased in ATRA-treated 5F8 (KO) cells and 2F5 (partially KO) cells compared with that in ATRA-treated WT cells. (I) Quantification of CD11b^+^ cells in ATRA-treated WT, 5F8 (KO), and 2F5 (partial KO) cells shown in (H). (J) Western blot shows LRRC25 was restored in KO (5F8) cells. (K) NBT reduction assay shows impaired ability of NBT reduction in LRRC25 knockout cells compared with WT cells under ATRA treatment, and NBT reduction ability of KO cells was rescued after restoration of LRRC25 under ATRA-treatment. (L) Quantification of NBT^+^ cells in ATRA-treated KO cells with or without restoration of LRRC25 shown in (K). All experiments were repeated at least 3 times, data of one representative experiment is shown. Student’s *t* test was used for statistics analysis. Error bar represents SD. **P < 0.01, ***P < 0.001

To elucidate the function of LRRC25 in ATRA-mediated granulocytic differentiation, we chose NB4 cells as a cell model for granulocytic differentiation. Initially, we used siRNA to knockdown expression of LRRC25 in NB4 cells (Fig. 5A). Figure 5B shows that NBT^+^ cells decreased significantly in the siLRRC25 group compared with those in the siNC group, demonstrating that LRRC25 knockdown impaired ATRA-induced terminal granulocytic differentiation. Then, we established NB4 cells by lentiviral methods, which stably knocked down LRRC25 (Fig. 5C), and confirmed that knockdown of LRRC25 expression significantly impaired ATRA-mediated granulocytic differentiation; NBT^+^ cells decreased significantly in the shLRRC25 group compared with those in the shN control group (Fig. 5D).

To assess the effect of *LRRC25* depletion, we used a CRISPR/Cas9 strategy for genome editing to generate *LRRC25* knockout cells in the NB4 cell line, which has human hypertriploid karyotype with 3% polyploidy. We confirmed the sequence of the gRNA targeting site [chr19 (−1): 18507682] in the NB4 cell line by Sanger sequencing, which suggested this cell line harbored WT sequences. Five positive cell clones derived from different single cells were screened from 56 clones. Sequencing analysis of PCR products covering gRNA-targeted site of these five clones showed non-WT genotypes, indicating a total of 6 mutants by T-transformant sequencing. Among these mutants, most were single nucleotide or short fragment-indels, while long fragment-inserts derived from the backbone of the gRNA vector were also observed. The 5F8 sequence had four types of mutation, all of which resulted in frameshift mutations and followed stop codons (Fig. 5E and 5F). Then, we determined the knockout efficiency in the mutated cells by RT-PCR and Western blot analyses. After treatment with ATRA, all clones expressed high levels of LRRC25 mRNA (These sequences may be mutated; 4G2 and 5B10 were used as WT controls randomly selected from all sequenced clones with WT sequence), demonstrating that all these clones were ATRA-responsive (Fig. S3), while Western blot results showed that LRRC25 was knocked out in the 5F8 clone (Fig. 5G).

In addition to NBT reduction, CD11b is also a marker of granulocytic differentiation (Atashrazm et al., 2016). Next, we treated WT NB4 cells, LRRC25 knockout cells (5F8) and partially knockout cells (2F5) with ATRA and evaluated the effect of LRRC25 depletion on granulocytic differentiation. Figure 5H and 5I show decreased CD11b^+^ cells in ATRA-treated 5F8 and 2F5 cells compared with those of WT cells. This result demonstrated that ATRA-induced granulocytic differentiation was inhibited after LRRC25 knockout in a dose-dependent manner. To exclude off-target effect of Cas9, we did sequencing work with the 5F8 and 2F5 clones and found the same non-targeted sequences in predicted top4-scored off-target sites (3 mismatches) as WT NB4 cells. In addition, we restored LRRC25 expression in 5F8 (KO) cells (Fig. 5J) and tested whether restoration of LRRC25 could rescue its ability to differentiate under ATRA treatment, using NBT reduction assay which is a standard test to evaluate granulocytic differentiation. As shown in Figure 5 K and 5L, percentage of cells that could reduce nitroblue tetrazolium decreased in the knockout group compared with the WT group, under ATRA treatment, which is consistent with CD11b changes. We also found restoration of LRRC25 in knockout cells rescued ATRA-induced granulocytic differentiation, by increasing its ability to reduce nitroblue tetrazolium (Fig. 5K and 5L). Therefore, lack of LRRC25 instead of off-target effect of Cas9 in the knockout cells contributed to the impaired ability to differentiate under ATRA treatment.

## DISCUSSION

LDA brought order to a chaotic field of applying monoclonal antibodies to immunology (Zola and Swart, 2005). This is the basis for immunophenotyping (Maecker et al., 2012), and these surface molecules play important roles in multiple biological activities including cell differentiation and pathogenesis of hematopoietic malignancies (Li et al., 2015). These antigens can serve as diagnostic or research markers of differentiation and cell lineage and therapeutic targets (Laszlo et al., 2014). Thus, identification of novel potential leukocyte differentiation antigens will help improve the classification of immune cells and understanding of the immune system and may also have clinical application. We built a gene expression database, which provides a convenient way to view global differential gene expression across thousands of experimental conditions in immune cells and is a useful tool to analyze potential biomarkers. Through big data mining, we identified a novel potential leukocyte differentiation molecule, LRRC25. With high ARSs and MESs in PMNs, monocytes, moderate ARSs in macrophages and DCs, and low ARSs and negative MESs in lymphocytes such as T cells, B cells, and NK cells, LRRC25 was predicted to be a potential leukocyte differentiation antigen (Wang et al., 2015). This was confirmed by up-regulated expression during myeloid cell development with RNA-seq data and low GPL score in myeloid cells, which indicates a myeloid marker or CD molecule (Wang et al., 2016). In our experiments, it was highly expressed in mature myeloid cells including granulocytes and monocytes, lowly/intermediately expressed in B cells, while poorly expressed in T cells and almost all NK cells. It was up-regulated during cell differentiation, displaying features of a leukocyte differentiation antigen. Paper reported that LRRC25 mRNA was expressed in dendritic cells, granulocytes, monocytes, and B cells instead of T cells, and the expression level of LRRC25 in B cells was obviously lower than that in granulocytes or monocytes, which is consistent with our results. In addition, it was down-regulated after activation of MDDCs (monocyte-derived dendritic cells), granulocytes, and B cells (Rissoan et al., 2002). Data used in our ImmuSort database was composed of cells with different activation status, and some B cell samples had low/intermediate expression of LRRC25.

LRRC25 is highly expressed in mature myeloid cells and up-regulated during the terminal differentiation of granulocytes, suggesting a potential physiological role in granulocytic differentiation. In our ATRA-induced granulocytic differentiation model, knockdown or knockout of LRRC25 inhibited terminal granulocytic differentiation. One typical feature of AML is blockade of differentiation, which is the outcome of multiple dysregulated genes (De Kouchkovsky and Abdul-Hay, 2016). We found that LRRC25 was significantly down-regulated in bone marrow cells of AML patients compared with that of healthy people. This may contribute to the pathogenesis of AML. However, the underlying mechanism of LRRC25 down-regulation remains unclear. There is an atypical CpG island near the promoter of *LRRC25*, and treatment of AML cell lines with demethylation drugs up-regulated expression of LRRC25 (Fig. S4A and S4B), suggesting that epigenetic regulation plays a role in controlling LRRC25 expression, directly or indirectly.

Our work showed that LRRC25 has a key role in ATRA-induced terminal granulocytic differentiation as a lack of LRRC25 significantly attenuated NBT reduction in a dose-dependent manner and restoration of LRRC25 in knockout cells rescued the NBT reduction ability. Ectopic expression of LRRC25 did not promote spontaneous differentiation of NB4 cells, possibly due to the absence of co-effectors, such as a potential ligand, as pathogenesis of AML is the outcome of dysregulation of multiple genes. This is the first report indicating that LRRC25 is a key regulator of granulocytic differentiation downstream of ATRA, illustrating a signal trunk in ATRA mediated differentiation therapy. The detailed mechanism by which LRRC25 is up-regulated and functions requires further study. Given the structural feature of the LRRC25 protein, adaptor molecules such as Syk or PI3K and/or a ligand may be necessary for this process. We also found that LRRC25 is up-regulated in DMSO-induced granulocytic differentiation and vitD3-induced monocytic differentiation in HL60 cells (Fig. S4C), which is consistent with a previous report (Vukic et al., 2015). These results suggest a possible role of LRRC25 in myeloid differentiation that is not restricted to ATRA-induced granulocytic differentiation. Moreover, LRRC25 is a potential pathogen recognition receptor (PRR), with leucine-rich repeats as the structural basis for pathogen recognition, and ITAM/ITIM for signaling transmission. Given that LRRC25 is highly expressed in myeloid cells, it may be involved in immune responses such as infection and inflammation, in addition to myeloid cell development. Thus, the role of LRRC25 should be crucial and may be mediated through the “closet” ITIM, which is usually employed in promoting appropriate immune cell behavior, i.e., LRRC25 may regulate subtly myeloid cell development or inflammatory responses as an adjustable switch.

Surface molecule detection by flow cytometry and molecular detection by quantitative polymerase chain reaction have been used in many aspects of leukemia, such as diagnosis, classification, risk stratification, monitoring minimal residual disease and prognosis (De Kouchkovsky and Abdul-Hay, 2016 ;Testa and Lo-Coco, 2016; Rocha et al., 2016). Myeloid differentiation antigens such as CD33 are broadly expressed on AML blasts, and a therapeutic antibody against CD33 (gemtuzumab ozogamicin, GO) was used for treatment of CD33-positive AML (Laszlo et al., 2014). In contrast to CD33, LRRC25 is down-regulated in the bone marrow cells of AML patients, suggesting a potential application of LRRC25 in the diagnosis of AML. ATRA treatment up-regulates LRRC25 expression in APL blasts, whether it could be a response marker for ATRA-based differentiation therapy needs to be further studied.

## MATERIALS AND METHODS

### Bioinformatics analysis

Differential expression was analyzed using publically available data sets from the gene expression omnibus (GEO) and ImmuSort databases (Wang et al.,^2015^). Mouse gene expression was directly derived from ImmGen (Heng and Painter, 2008). Array-based data sets including GSE17054 (Majeti et al., 2009), GSE19599 (Andersson et al., 2010), and GSE28490 (Allantaz et al., 2012), were downloaded to calculate expression values based on a method described previously (Wang et al., 2015a, b). For RNA sequencing (RNA-seq) data, the data sets GSE69239 (Casero et al., 2015) and GSE25133 (Valouev et al., 2011) were used.

### Cell lines and primary leukemia cells

NB4, U937, K562, and MEG-01 cells were maintained in our laboratory. THP-1 and HL60 cells were obtained from the American Type Culture Collection (ATCC, USA). All cells were routinely grown in RPMI-1640 (Gibco BRL, USA) supplemented with 10% (*v*/*v*) heat-inactivated FBS (HyClone, USA). Bone marrow cells from healthy individuals and AML patients were obtained from the Institute of Hematology, Peking University People’s Hospital, with the patients’ consent and institutional ethics approval.

### siRNA and shRNA against LRRC25

The sense sequences of siRNAs against LRRC25 are provided below. These siRNAs were synthesized by GenePharma (Suzhou, China). si1361: 5′-CCGACUAUGAGAACAUGUUTT-3′; si1432: 5′-UCAGAGGACAAUGACUUUUTT-3′.

Mature antisense sequences of shRNAs against LRRC25 are provided below. The lentiviral plasmids pGIPZ-shLRRC25 used for shRNA knockdown were purchased from Thermo Fisher Scientific (Cat. RHS4531-EG126364. USA). sh899: 5′-TCTAGGCTGTCTGACTCCC-3′; sh903: 5′-AAAAGTCATTGTCCTCTGA-3′.

### RNA extraction, reverse transcription and semi-quantitative PCR, and real-time PCR

Extraction of total RNA and synthesis of single-stranded complementary DNA (cDNA) were performed as described previously. Human Multiple Tissue and Immune System MTC^TM^ panels containing cDNA from a pool of donors were purchased from Clontech (California, USA). The cDNA mixtures were amplified by PCR using LRRC25-specific forward (5′-GGTGCCTGCTTCTTGGACTTG-3′) and reverse (5′-AGGTCGATGTCCTTGTAGTTGATGT-3′) primers and 2× Taq PCR StarMix (GenStar, China). ACTB was used as an internal control and amplified with ACTB-specific forward (5′-GAGCACAGAGCCTCGCCTTT-3′) and reverse (5′-TTCATGAGGTAGTCAGTCAGGTCCC-3′) primers. Each amplification reaction underwent 26–28 cycles (LRRC25) or 22 cycles (ACTB) of denaturation at 95°C for 30 s, annealing for 30 s at 60°C (LRRC25) or 62°C (ACTB), and elongation at 72°C for 30 s. Real-time PCR reactions were performed using TaqMan probes (Roche, Switzerland) and an Eco Real-Time PCR System (Illumina Inc., USA). The sequences of the LRRC25 primers were as follows: forward, 5′-CCCTCCACTCCCGACTATGAG-3′ and reverse, 5′-TGTCCTCTGAAGGGTGAGCC-3′. The sequences of ACTB primers were as follows: forward, 5′-CCAACCGCGAGAAGATGA-3′ and reverse, 5′-CCAGAGGCGTACAGGGATAG-3′.

### Preparation of antibodies

Rabbit anti-LRRC25 polyclonal antibody was generated using a prokaryotic fusion protein of the intracellular C-terminus of LRRC25 tagged with GST, namely, GST-LRRC25ic, as an immunogen. Primers used for construction of plasmids were as follows: LRRC25ic-F/R, 5′-GAAGAATTCCTGGAGACTCTGGCGATGCC-3′ and 5′-GTTGTTGCGGCCGCTTAGTGCCCGGGGATCAC-3′). Transformed bacteria were cultured with 0.1 mmol/L IPTG at 16°C for 20 h. Purification of the GST-LRRC25ic protein and preparation of rabbit anti-LRRC25 polyclonal antibody were performed as described (Li et al., 2012).

Mouse anti-LRRC25 monoclonal antibodies were prepared using the eukaryotic fusion protein of extracellular N-terminus of LRRC25 tagged with Fc, namely, LRRC25ec-Fc, as an immunogen. Primers used for construction of plasmid were as follows: LRRC25ec-F/R, 5′-CGGAATTCAGCCTAGAACCGTCGTGC-3′ and 5′-GCTCTAGAACCTCCACCAGTTGCAGAGGCCAGGCC-3′. Akesobio (Guangdong, China) produced the recombinant eukaryotic proteins, LRRC25ec-Fc and Fc, and Beijing Protein Innovation Co., Ltd (Beijing, China) generated the mouse anti-LRRC25 monoclonal antibodies.

### Electroporation

For localization analysis, 3 × 10^6^ HeLa cells were mixed with 15 μg of plasmids (pEGFP-N1 or pEGFP-N1-LRRC25) and electroporated at 120 V for 20 ms in a BTX machine (Harvard Apparatus, USA). NB4 cells were electroporated with the SF cell line 4D Nucleofector X Kit (V4XC-2024) in a LONZA 4DX machine (LONZA, Germany). Briefly, 2 × 10^6^ cells were mixed with 4.5 μg of pX330-GFP-Cas9-gRNA-LRRC25 or siRNA (at a final concentration of 20 nmol/L) in 82 μL of SF solution supplied with 18 μL of supplement and electroporated under a program of CZ-100.

### Restored expression of LRRC25 in NB4 cells and establishment of NB4 cells stably transfected with pGIPZ-shLRRC25

To restore expression of LRRC25, we constructed the lentiviral vector pLenti6.3/V5-TOPO-LRRC25. The primers used were as follows: forward, 5′-CGCGGATCCACCATGGGGGGCACCC-3′; reverse, 5′-TCCGCTCGAGCGTCAGTGCCCGGGGATCACGTAC-3′. Lentiviral particles were packaged using pLenti6.3/V5-TOPO or pLenti6.3/V5-TOPO-LRRC25, psPAX2, and pHCMV-G. NB4 cells or LRRC25 knockout 5F8 clone were infected with supernatants containing lentivirus with polybrene in 6-well plate at 16°C at 500 ×*g* for 30 min. Infected cells were selected with 10 μg/mL of blasticidin S until all wild-type (WT) NB4 cells died.

NB4 cells were infected with supernatants containing lentivirus packaged using pGIPZ-shN or pGIPZ-shLRRC25, psPAX2, and pHCMV-G, as described above. Infected cells were selected with 1 μg/mL of puromycin until all WT NB4 cells died. LRRC25 could be stably knocked down in these cells when it was up-regulated by ATRA.

### Knockout of LRRC25 in NB4 cells with CRISPR-Cas9 technology

Shanghai Biomodel Organism Science & Technology Development Co.,Ltd. (China) prepared the pX330-GFP-Cas9-gRNA-LRRC25 plasmid containing guide RNA (gRNA) sequences targeting LRRC25. The targeting sequence of gRNA was 5′-GTCGTGCACCGTGTCCTCCG-3′ with a protospacer adjacent motif (PAM) of CGG. NB4 cells were electroporated with the pX330-GFP-Cas9-gRNA-LRRC25 plasmid. Four days later, GFP+ cells were sorted into 96-well plates (single cell per well) using a BD FACS SORP system. Cells from different clones were collected to prepare genomic DNA with a genomic DNA extraction kit (Aidlab, China). We amplified fragments containing the targeting site of LRRC25 with PrimeSTAR^®^ HS DNA Polymerase (TaKaRa, Japan) (forward: 5′-GGTTAACTGGCCCGGATCTC-3′ and reverse: 5′-CTGGTTGTGTCCCAGCAGAG-3′). The forward primer was also used for sequencing analysis. Clones with mutant genotype were selected as positive clones. For verification of the sequence of targeted sites, PCR products were ligated into pBLUE-T vectors and sequenced. Off-target effects were eliminated by Sanger sequencing. Primers used for the PCR reactions were as follows: Offtarget1-F (5′-TTTTCCCATGACCTCACCCG-3′) and R (5′-CTGGAGACCAGGGAGGAGAG-3′); Offtarget2-F (5′-GCCTTGCAGTGGTAGCTGAA-3′) and R (5′-GAGGGCTGGTGGGTATCTTG-3′); Offtarget3-F (5′-CGTCCACTGTGCAGCTTGTA-3′) and R (5′-CCCTCCTGTTGGGTAAGGGT-3′); Offtarget4-F (5′-GATCTTCTCCTGCACCGTGG-3′) and R (5′-TGTGGAGAAGGAGGTGGAGA-3′). Clones with specific mutations that were confirmed by other strategies were used for further functional analysis.

### Western blot

Cells were harvested and washed twice in cold PBS and lysed in NP40 lysis buffer (50 mmol/L Tris-HCl, 150 mmol/L NaCl, 0.5% NP40, pH 7.5) supplemented with protease inhibitors (complete Mini EDTA-free protease inhibitor mixture tablets; Roche, Germany) for 30 min on ice and centrifuged at 4°C at 12,000 ×*g* for 10 min to remove debris. Total proteins in supernatants were quantified with a BCA kit (Thermo Fisher Scientific, USA). Samples were added with loading buffer (consists of β-mercaptoethanol and SDS), denatured at 99°C for 10 min and equilibrated to room temperature, and then 50 or 100 μg of proteins were subjected to 12.5% SDS-PAGE and transferred to a nitrocellulose blotting membrane with a Pyxis machine (GE Healthcare, USA). Rabbit anti-LRRC25ic polyclonal antibody (1 μg/mL) was used to detect LRRC25, β-actin was detected with a mouse anti-human β-actin (1:3,000 dilution, Clone AC-74, Sigma, USA) as a loading control, and secondary antibodies were from Cell Signaling Technology (USA), including HRP-conjugated goat anti-rabbit IgG antibody and goat anti-mouse IgG antibody (1:5,000 dilution). Signals were detected by enhanced chemiluminescence (ECL) Western blot detection reagents (GE, Healthcare) on an ImageQuant^TM^ LAS500 (GE Healthcare, USA).

### Confocal microscopy

HeLa cells transfected with pEGFP-N1 or pEGFP-N1-LRRC25 adhered to 24-well slides. After 48 h, the cells were fixed with 2% PFA, permeabilized with 0.5% Triton X-100, and stained with Hoechst 33342 (Sigma, USA). Stained cells were visualized using an Olympus FV10i fluorescence confocal microscope (Olympus, USA). NB4 cells treated with 1 μmol/L ATRA for 4 days were cytospun on slide glasses, fixed, and incubated with mouse anti-LRRC25ec monoclonal antibody (No. 70) or mouse IgG1 isotype control antibody (Sigma, Cat. No. M7894) for 1 h. Then, the cells were washed and incubated with FITC-conjugated goat anti-mouse IgG for 30 min. The cells were washed with PBS, permeabilized with 0.5% Triton X-100, and stained with Hoechst 33342. Photograph of stained cells were taken by confocal microscopy as described above.

### Analysis of LRRC25 expression on primary peripheral leukocytes and NB4 cells

Fresh peripheral blood was obtained from healthy donors. Peripheral mononuclear cells (PBMCs) and granulocytes fractions were isolated by Ficoll-Hypaque density gradient centrifugation. PBMCs were used for T cells, B cells, NK cells, and monocytes staining of LRRC25 expression, and granulocytes fraction was used for neutrophil staining after lysing red cells. 1 × 10^6^ cells were used per test for flow cytometric analysis. The cells were resuspended and blocked for 20 min in 100 μL of blocking buffer (5% heat-inactivated FBS in PBS supplied with 20 μL of human FcR inhibitor, Affymetrix eBioscience, Cat. 14-9161) followed by incubation with 2 μg of mouse anti-human LRRC25 monoclonal antibody (mAb70) or mouse IgG1, κ (M5284, Sigma) on ice for 1 h. Free primary antibody was washed with cold PBS, and cells were incubated on ice with FITC goat anti-mouse IgG antibody (ZF-0312, ZSGB-BIO) in dark for 30 min. Free secondary antibody was washed with cold PBS and cells were incubated on ice with PerCP mouse anti-human CD3 (300428, BioLegend, for T cells), PerCP mouse anti-human CD3 and APC mouse anti-human CD19 (302212, BioLegend) (for B cells), PerCP mouse anti-human CD3 and APC mouse anti-human CD56 (318309, Biolegend) (for NK cells), PE mouse anti-human CD14 (555398, BD Pharmingen^TM^) (for monocytes), PE mouse anti-human CD11b (555388, BD Pharmingen^TM^), and APC mouse anti-human CD16 (302011, BioLegend) (for neutrophils) in dark for 30 min. Free marker antibodies were washed with cold PBS, then the cells were resuspended in 200 μL of cold PBS and analyzed in BD FACSVerse^TM^. PerCP mouse IgG (559425), APC mouse IgG (555751), PE mouse IgG (555749) were from BD Pharmingen^TM^ and used as isotype control antibodies. After being gated based on the forward and side scatter, B cells and NK cells were gated on CD3^−^ cell population and analyzed for co-expression of LRRC25 with CD19 or CD56, neutrophils were gated on CD11b^+^ cell population and analyzed for co-expression of LRRC25 with CD16. Quadrants were generated based on the staining of isotype antibodies so that isotype control- stained cells were in the bottom part of the quadrant. Flow cytometric analysis of LRRC25 expression in NB4 cells was done as described above. Samples were analyzed in BD FACSVerse^TM^.

### CCK8 assay and cell counting assay

For CCK8 assays, 3,000 cells per well were plated in 96-well plate, and 6 wells were used for each time point. Then, 10 μL of CCK8 reagent (Dojindo, Tokyo) was added in each well, and the cells were incubated for 3 h, followed by analysis with a Thermo Fisher Scientific Multiskan GO. For cell counting assays, cells were plated at a cell density of 3 × 10^4^/mL, cultured for 6 days and counted every two days using a blood counting chamber (Shanghai, China).

### Differentiation of leukemia cells and detection of differentiation

NB4 and HL60 cells were treated with 1 μmol/L ATRA (Sigma, USA) for 4 days to induce granulocytic differentiation. Bone marrow cells from APL patients were treated with 1 μmol/L ATRA for 7 days for granulocytic differentiation. For siRNA knockdown assays, NB4 cells were electroporated with siLRRC25, and 6 h later, the cells were treated with 1 μmol/L ATRA for 48 h. For shRNA knockdown assays, NB4 cells stably transfected with pGIPZ-shLRRC25 were treated with 1 μmol/L ATRA for 96 h. For LRRC25 knockout assays, knockout cells or KO cells with restored LRRC25 were treated with 1 μmol/L ATRA for 96 h.

CD11b on the cell surface was detected by direct immunofluorescence staining. Briefly, cells were collected and washed with cold PBS, blocked with 2% FBS for 10 min on ice, incubated with PE-mouse anti-human CD11b (BD PMG, Cat. No. 555388) or PE-mouse IgG isotype control antibody (BD PMG, Cat. No. 555749) on ice for 30 min in the dark, washed and resuspended in PBS for FACS analysis in a BD FACSCalibur™ or with a FlowJo software. Nitroblue tetrazolium (NBT) reduction assays were carried out as described previously (Nishioka et al. 2008).

### Statistical analysis

Experiments were repeated at least 3 times unless otherwise indicated. Student’s *t* test was used for statistical analysis. Statistical significance was set at a *P* value of less than 0.05 (*P < 0.05, **P < 0.01, ***P < 0.001, n.s. represents no significance).

## Electronic supplementary material

Below is the link to the electronic supplementary material.

Supplementary material 1 (PDF 375 kb)

## References

[CR1] Allantaz F, Cheng DT, Bergauer T, Ravindran P, Rossier MF, Ebeling M, Badi L, Reis B, Bitter H, D’Asaro M, Chiappe A, Sridhar S, Pacheco GD, Burczynski ME, Hochstrasser D, Vonderscher J, Matthes T (2012). Expression profiling of human immune cell subsets identifies miRNA-mRNA regulatory relationships correlated with cell type specific expression. PLoS ONE.

[CR2] Andersson A, Eden P, Olofsson T, Fioretos T (2010). Gene expression signatures in childhood acute leukemias are largely unique and distinct from those of normal tissues and other malignancies. BMC Med Genom.

[CR3] Atashrazm F, Lowenthal RM, Dickinson JL, Holloway AF, Woods GM (2016). Fucoidan enhances the therapeutic potential of arsenic trioxide and all-trans retinoic acid in acute promyelocytic leukemia, in vitro and in vivo. Oncotarget.

[CR4] Barrow AD, Trowsdale J (2006). You say ITAM and I say ITIM, let’s call the whole thing off: the ambiguity of immunoreceptor signalling. Eur J Immunol.

[CR5] Casero D, Sandoval S, Seet CS, Scholes J, Zhu Y, Ha VL, Luong A, Parekh C, Crooks GM (2015). Long non-coding RNA profiling of human lymphoid progenitor cells reveals transcriptional divergence of B cell and T cell lineages. Nat Immunol.

[CR6] Cicconi L, Lo-Coco F (2016). Current management of newly diagnosed acute promyelocytic leukemia. Ann Oncol.

[CR7] De Braekeleer E, Douet-Guilbert N, De Braekeleer M (2014). RARA fusion genes in acute promyelocytic leukemia: a review. Expert Rev Hematol.

[CR8] De Kouchkovsky I, Abdul-Hay M (2016). Acute myeloid leukemia: a comprehensive review and 2016 update. Blood Cancer J.

[CR9] Dos SG, Kats L, Pandolfi PP (2013). Synergy against PML-RARa: targeting transcription, proteolysis, differentiation, and self-renewal in acute promyelocytic leukemia. J Exp Med.

[CR10] Gaillard C, Tokuyasu TA, Rosen G, Sotzen J, Vitaliano-Prunier A, Roy R, Passegue E, de The H, Figueroa ME, Kogan SC (2015). Transcription and methylation analyses of preleukemic promyelocytes indicate a dual role for PML/RARA in leukemia initiation. Haematologica.

[CR11] Heng TS, Painter MW (2008). The Immunological Genome Project: networks of gene expression in immune cells. Nat Immunol.

[CR12] Idaghdour Y, Quinlan J, Goulet JP, Berghout J, Gbeha E, Bruat V, de Malliard T, Grenier JC, Gomez S, Gros P, Rahimy MC, Sanni A, Awadalla P (2012). Evidence for additive and interaction effects of host genotype and infection in malaria. Proc Natl Acad Sci U S A.

[CR13] Jasek E, Mirecka J, Litwin JA (2008). Effect of differentiating agents (all-trans retinoic acid and phorbol 12-myristate 13-acetate) on drug sensitivity of HL60 and NB4 cells in vitro. Folia Histochem Cytobiol.

[CR14] Kedzierski L, Montgomery J, Curtis J, Handman E (2004). Leucine-rich repeats in host-pathogen interactions. Arch Immunol Ther Exp (Warsz).

[CR15] Laszlo GS, Estey EH, Walter RB (2014). The past and future of CD33 as therapeutic target in acute myeloid leukemia. Blood Rev.

[CR16] Li T, Guo XH, Wang PZ, Song QS, Ma DL, Han WL (2012). Preparation and identification of the polyclonal antibody against human VSTM1. Xi Bao Yu Fen Zi Mian Yi Xue Za Zhi.

[CR17] Li T, Guo X, Wang W, Mo X, Wang P, Han W (2015). Vset and transmembrane domaincontaining 1 is silenced in human hematopoietic malignancy cell lines with promoter methylation and has inhibitory effects on cell growth. Mol Med Rep.

[CR18] Ma W, Gilligan BM, Yuan J, Li T (2016). Current status and perspectives in translational biomarker research for PD-1/PD-L1 immune checkpoint blockade therapy. J Hematol Oncol.

[CR19] Maecker HT, McCoy JP, Nussenblatt R (2012). Standardizing immunophenotyping for the Human Immunology Project. Nat Rev Immunol.

[CR20] Majeti R, Becker MW, Tian Q, Lee TL, Yan X, Liu R, Chiang JH, Hood L, Clarke MF, Weissman IL (2009). Dysregulated gene expression networks in human acute myelogenous leukemia stem cells. Proc Natl Acad Sci U S A.

[CR21] Nishioka C, Ikezoe T, Yang J, Koeffler HP, Yokoyama A (2008). Blockade of mTOR signaling potentiates the ability of histone deacetylase inhibitor to induce growth arrest and differentiation of acute myelogenous leukemia cells. Leukemia.

[CR22] Nishioka C, Ikezoe T, Yang J, Gery S, Koeffler HP, Yokoyama A (2009). Inhibition of mammalian target of rapamycin signaling potentiates the effects of all-trans retinoic acid to induce growth arrest and differentiation of human acute myelogenous leukemia cells. Int J Cancer.

[CR23] Nitto T, Sawaki K (2014). Molecular mechanisms of the antileukemia activities of retinoid and arsenic. J Pharmacol Sci.

[CR24] Rissoan MC, Duhen T, Bridon JM, Bendriss-Vermare N, Peronne C, de Saint VB, Briere F, Bates EE (2002). Subtractive hybridization reveals the expression of immunoglobulin-like transcript 7, Eph-B1, granzyme B, and 3 novel transcripts in human plasmacytoid dendritic cells. Blood.

[CR25] Rocha JM, Xavier SG, de Lima SM, Assumpcao JG, Murao M, de Oliveira BM (2016). Current Strategies for the Detection of Minimal Residual Disease in Childhood Acute Lymphoblastic Leukemia. Mediterr J Hematol Infect Dis.

[CR26] Testa U, Lo-Coco F (2016). Prognostic factors in acute promyelocytic leukemia: strategies to define high-risk patients. Ann Hematol.

[CR27] Valouev A, Johnson SM, Boyd SD, Smith CL, Fire AZ, Sidow A (2011). Determinants of nucleosome organization in primary human cells. Nature.

[CR28] Vukic M, Neme A, Seuter S, Saksa N, de Mello VD, Nurmi T, Uusitupa M, Tuomainen TP, Virtanen JK, Carlberg C (2015). Relevance of vitamin D receptor target genes for monitoring the vitamin D responsiveness of primary human cells. PLoS ONE.

[CR29] Wang ZY, Chen Z (2008). Acute promyelocytic leukemia: from highly fatal to highly curable. Blood.

[CR30] Wang P, Yang Y, Han W, Ma D (2015). ImmuSort, a database on gene plasticity and electronic sorting for immune cells. Sci Rep.

[CR31] Wang P, Qi H, Song S, Li S, Huang N, Han W, Ma D (2015). ImmuCo: a database of gene co-expression in immune cells. Nucleic Acids Res.

[CR32] Wang P, Han W, Ma D (2016). Electronic sorting of immune cell subpopulations based on highly plastic genes. J Immunol.

[CR33] Zola H, Swart B (2005). The human leucocyte differentiation antigens (HLDA) workshops: the evolving role of antibodies in research, diagnosis and therapy. Cell Res.

