# Supplementary material for: LRRC25 plays a key role in all-trans retinoic acid-induced granulocytic differentiation as a novel potential leukocyte differentiation antigen
Source: Protein Cell. 2017 May 23;9(9):785–98. doi: 10.1007/s13238-017-0421-7 (PMC6107485; doi:10.1007/s13238-017-0421-7)
Supplement: Supplementary file 1 — Supplementary material 1 (PDF 375 kb) [file 13238_2017_421_MOESM1_ESM.pdf]

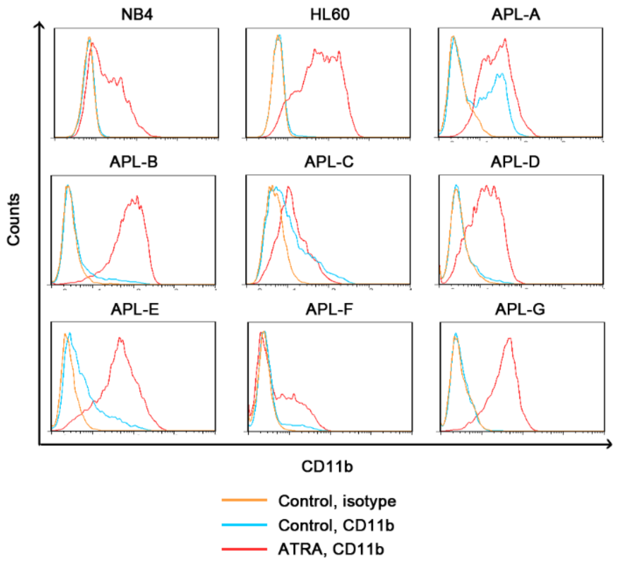

Figure S1: CD11b expression by flow cytometry was shown to indicate differentiation of AML cell lines and bone marrow cells of APL patients before and after ATRA-treatment.

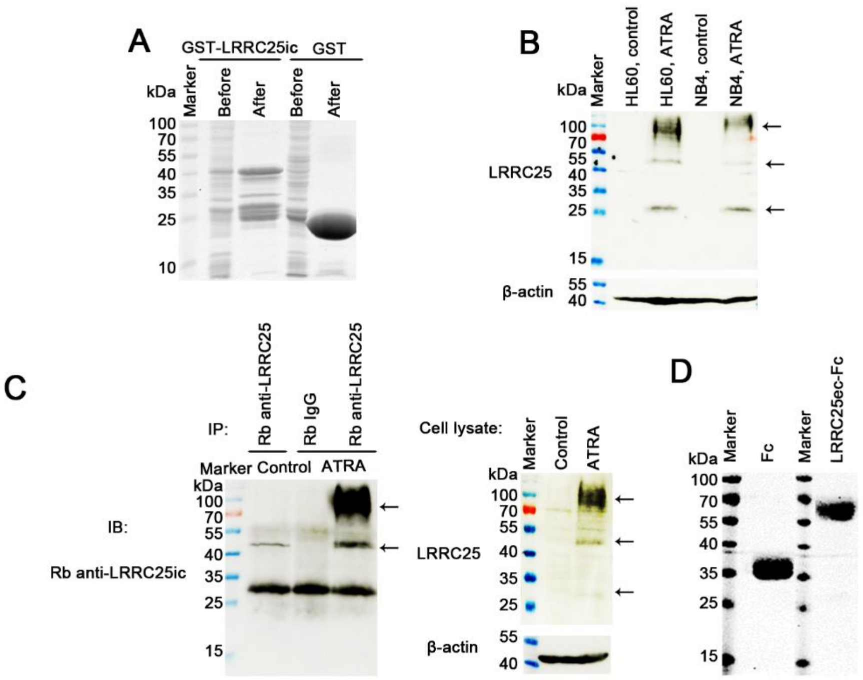

Figure S2: Preparation of prokaryotic and eukaryotic proteins and identification of rabbit anti-LRRC25ic polyclonal antibody. A, Identification of GST-LRRC25ic and

GST protein before and after purification. B, Western blot shows the rabbit anti-human LRRC25ic polyclonal antibody recognized three specific bands as indicated by arrows. C, Rabbit anti-LRRC25ic polyclonal antibody could be used for immunoprecipitation of intrinsic LRRC25 in ATRA-treated NB4 cells (left panel), right panel shows 10% of input. D. Identification of purified Fc and LRRC25ec-Fc recombinant proteins.

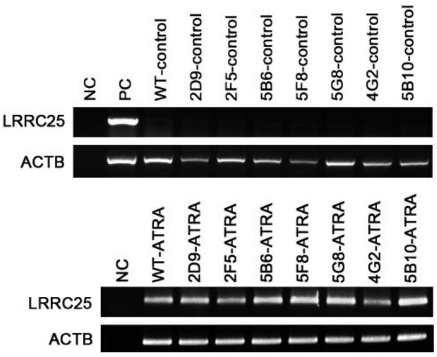

Figure S3: Semi-quantitative PCR shows all CRISPR-Cas9 targeted clones responded to ATRA with LRRC25 mRNA expression (with or without mutation). 4G2 and 5B10 were used as two randomly selected WT control clones.

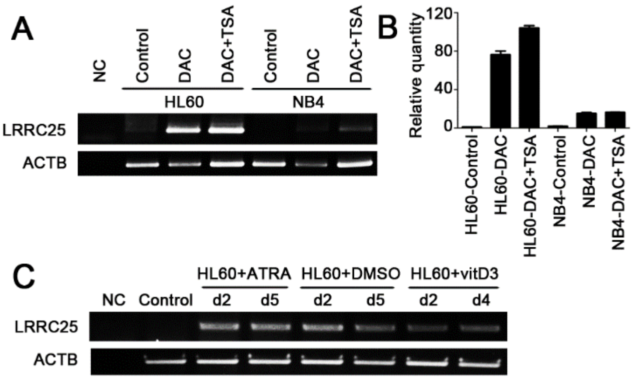

Figure S4: LRRC25 could be regulated by epigenetic drugs and up-regulated by other inducing reagents. A and B, Semi-quantitative and real-time PCR show LRRC25 was

up-regulated in AML cells when treated with 5-aza-2’-deoxycytidine (DAC) or combination of DAC and trichostatin A (TSA), which are demethylation drug and histone deacetylase inhibitor, respectively. HL60 was treated with 500nM DAC for 3 days or with 200nM TSA for the last 1 day. Data in triplicates were shown in mean ± SD in S4B. C, Semi-quantitative PCR shows LRRC25 could be up-regulated with ATRA (1 μM), DMSO (1.25%) and vitD3 (100 nM) in HL60 cells.
